# Influence of generic drug supply restrictions on quetiapine tablet dispensing ratio and medication costs in community pharmacies in Japan

**DOI:** 10.1186/s12913-026-15250-2

**Published:** 2026-07-29

**Authors:** Erisa Mukai, Yuki Kondo, Tomoko Kondo, Naoto Okada, Azusa Miura, Kayoko Kubota, Hirokazu Azuma, Shinichiro Yamakado, Yoichi Ishitsuka

**Affiliations:** 1 Minaminihon Pharmaceutical Center, 5-15-1 Taniyama-chuo, Kagoshima, 891-0141 Japan; 2 Department of Clinical Chemistry and Informatics, Graduate School of Pharmaceutical Sciences, Kumamoto University, 5-1 Oe-honmachi, Chuo- ku, Kumamoto, 862-0973 Japan; 3 Joint Research Center for Human Retrovirus Infection, Kagoshima University, 8-35-1, Sakuragaoka, Kagoshima 890-8544 Japan; 4 Clinical Pharmacology, Graduate School of Medicine, Yamaguchi University, 1-1-1 Minamikogushi, Ube, Yamaguchi 755-8505 Japan; 5 Pharmacy Department, Yamaguchi University Hospital, 1-1-1 Minamikogushi, Ube, Yamaguchi 755-8505 Japan

**Keywords:** Generic drugs, Supply restrictions, Generic dispensing ratio, Medication costs, Quality

## Abstract

**Background:**

To limit health care expenditures and alleviate the burden on consumers, maintaining a stable supply of generic pharmaceuticals has become a major health care challenge. Recently, suspensions in the distribution of generic drugs and supply restrictions for pharmaceuticals with the same active ingredients have occurred owing to quality test alterations by pharmaceutical companies and shortages of drug ingredients during the coronavirus disease 2019 (COVID-19) pandemic. This study investigated changes in the generic dispensing ratio and medication costs for quetiapine tablets before and after supply restrictions to evaluate their influence on pharmaceutical supply.

**Methods:**

This retrospective observational study used routinely collected dispensing data from nine community pharmacies operated by Minaminihon Pharmaceutical Center. We used a non-probability sampling approach and included all eligible prescriptions containing quetiapine tablets dispensed at the participating pharmacies from May to December 2021. The daily generic dispensing ratio and medication costs for quetiapine tablets were compared between the periods before and after the supply restrictions. September 1, 2021, was defined as the intervention point, and changes in these outcomes associated with the supply restrictions were assessed using interrupted time-series (ITS) analysis.

**Results:**

The generic dispensing ratio was significantly lower after the supply restrictions than before the restrictions (before: 95.53%; after: 67.19%; *p* < 0.0001). The median medication cost per patient per day increased significantly from 14.4 Japanese yen (JPY; approximately 0.13 US dollars [USD]/0.11 euros [EUR]) before the supply restrictions to 20.2 JPY (approximately 0.18 USD/0.16 EUR) afterward (*p* < 0.01). In the ITS analysis using a generalized linear model, the slope changes after the supply restrictions were − 0.0077 for the generic dispensing ratio (95% confidence interval [CI]: −0.0120 to − 0.0034; *p* = 0.0004) and 0.0043 for daily medication costs per prescription (95% CI: 0.0035 to 0.0051; *p* < 0.0001), respectively.

**Conclusions:**

Generic drug supply restrictions for quetiapine tablets were associated with a decrease in the generic dispensing ratio and an increase in medication costs. The ITS analysis suggested that these effects became more pronounced over time after the start of supply restrictions.

## Background

In Japan, medical expenses have been steadily increasing owing to the introduction of high-cost medications, advances in medical technology, and population aging. Therefore, as part of the medical expenses reduction policy, the Ministry of Health, Labour and Welfare has been taking initiatives, such as establishing the “Roadmap for Further Promotion of Generic Drugs” in April 2013 [1]. According to the Ministry of Health, Labour and Welfare, the usage rate of generic drugs (calculated as quantity of generic drugs/quantity of branded drugs including generics+quantity of generic drugs) has increased from 46.9% as of September 2013 to 79.0% as of September 2022 [2].

Amidst the implementation of these generic drug promotion policies, in December 2020 it was revealed that an ingredient in a sleep medication had been mixed into an antifungal drug manufactured by a generic drug company. This incident triggered a series of revelations involving problems such as tampering with quality tests by pharmaceutical companies (Table 1), leading to the supply suspensions of generic drugs and restrictions in the supply of drugs with the same active ingredients as those in drugs manufactured by other companies.

Restrictions in the supply of generic drugs may lead to a decline in generic drug use and an increase in the financial burden on patients. Quetiapine is an antipsychotic medication used in routine clinical practice, and multiple generic quetiapine tablet products have been dispensed in community pharmacies in Japan. During periods of generic drug supply instability, supply restrictions for quetiapine tablets may require pharmacies to change dispensed products depending on availability, including switching from generic to branded products. Such changes may reduce the generic dispensing ratio and increase medication costs. However, evidence on how generic drug supply restrictions affect dispensing practices and medication costs in community pharmacies remains limited. Therefore, this study aimed to evaluate the influence of generic drug supply restrictions on the generic dispensing ratio and medication costs for quetiapine tablets in community pharmacies in Japan. To assess changes in these outcomes before and after the start of supply restrictions, we conducted an interrupted time-series (ITS) analysis in a quasi-experimental design.

**Table 1:** Recent administrative penalties (cessation or improvement of business orders) in pharmaceutical companies of Japan

Date	Company [Office location]	Cessation or Improvement of business orders	Reasons
2021.2.9	Kobayashi Kako Co., Ltd. [Fukui]	Cessation and improvement	A production procedure of the medicine is different from an approval book
2021.3.5	Nichi-Iko Pharmaceutical Co., Ltd. [Toyama]	Cessation	A production procedure of the medicine is different from an approval book
2021.4.28	Kobayashi Kako Co., Ltd. [Fukui]	Improvement	Misstatements in application documents
2021.5.21	Meiji Seika Pharma Co., Ltd. [Tokyo], Elmed Co., Ltd. [Toyama] and Daiichi Sankyo Espha Co., Ltd. [Tokyo]	Improvement	Misstatements in application documents (Joint with Kobayashi Kako Co.,Ltd.)
2021.3.27	Okami Chemical Industry Co., Ltd. [Kyoto]	Cessation and improvement	Defects such as manufacturing management, quality control system, and manufacturing facility
2021.8.12	Hisamitsu Pharmaceutical Co., Ltd. [Saga]	Cessation	Production of the medicine using a nonstandard coloring agent
2021.9.14	Kitanihon Pharmaceutical Co., Ltd. [Toyama]	Cessation and improvement	A production procedure of the medicine is different from an approval book
2021.10.11	Choseido Pharmaceutical Co., Ltd. [Tokushima]	Cessation and improvement	A production procedure of the medicine is different from an approval book
2021.11.12	Matsuda Chemical Industries, Ltd. [Ehime]	Cessation and improvement	A production procedure of the medicine is different from an approval book
2021.12.24	Nissin Pharmaceutical Co., Ltd. [Shiga]	Cessation and improvement	An ingredient and quantity of the medicine is different from an approval book
2022.1.19	Fuji Pharma Co., Ltd. [Toyama]	Improvement	Partial non-enforcement for purity tests

## Methods

### Study design and data source

This was a retrospective observational study using routinely collected dispensing data extracted from the dispensing information database of nine community pharmacies operated by Minaminihon Pharmaceutical Center. We used an ITS design to evaluate changes in the generic dispensing ratio and medication costs for quetiapine tablets before and after the start of generic drug supply restrictions.

### Study population, eligibility criteria, and sampling approach

The study population comprised prescriptions for patients who were dispensed quetiapine tablets, including 12.5-mg, 25-mg, and 100-mg tablets, between May 1 and December 31, 2021. Prescriptions were eligible for inclusion if they contained quetiapine tablets dispensed at one of the participating pharmacies during the study period and had the information required to determine the product type, strength, dosage, prescription duration, and medication cost. We used a non-probability sampling approach based on routinely collected dispensing data from the participating community pharmacies. Within this data source, all eligible prescriptions for quetiapine tablets dispensed during the study period were included in the analysis. The extracted dispensing data included information on the product type, strength, dosage, prescription duration, and medication cost. The dataset was anonymized before analysis and did not include patient-level demographic or clinical information, such as age, sex, diagnosis, reasons for quetiapine use, occupation, education status, or family background.

### Outcomes and intervention point

We compared the daily generic dispensing ratio and medication costs before and after the supply restrictions. The generic dispensing ratio was defined as the number of prescriptions dispensed with generic quetiapine tablets divided by the total number of prescriptions including quetiapine tablets each day. September 1, 2021, was defined as the intervention point because supply restrictions for quetiapine tablets began in September 2021. The pre-restriction period was from May 1 to August 31, 2021, and the post-restriction period was from September 1 to December 31, 2021.

### Statistical analysis

**Fig. 1:**
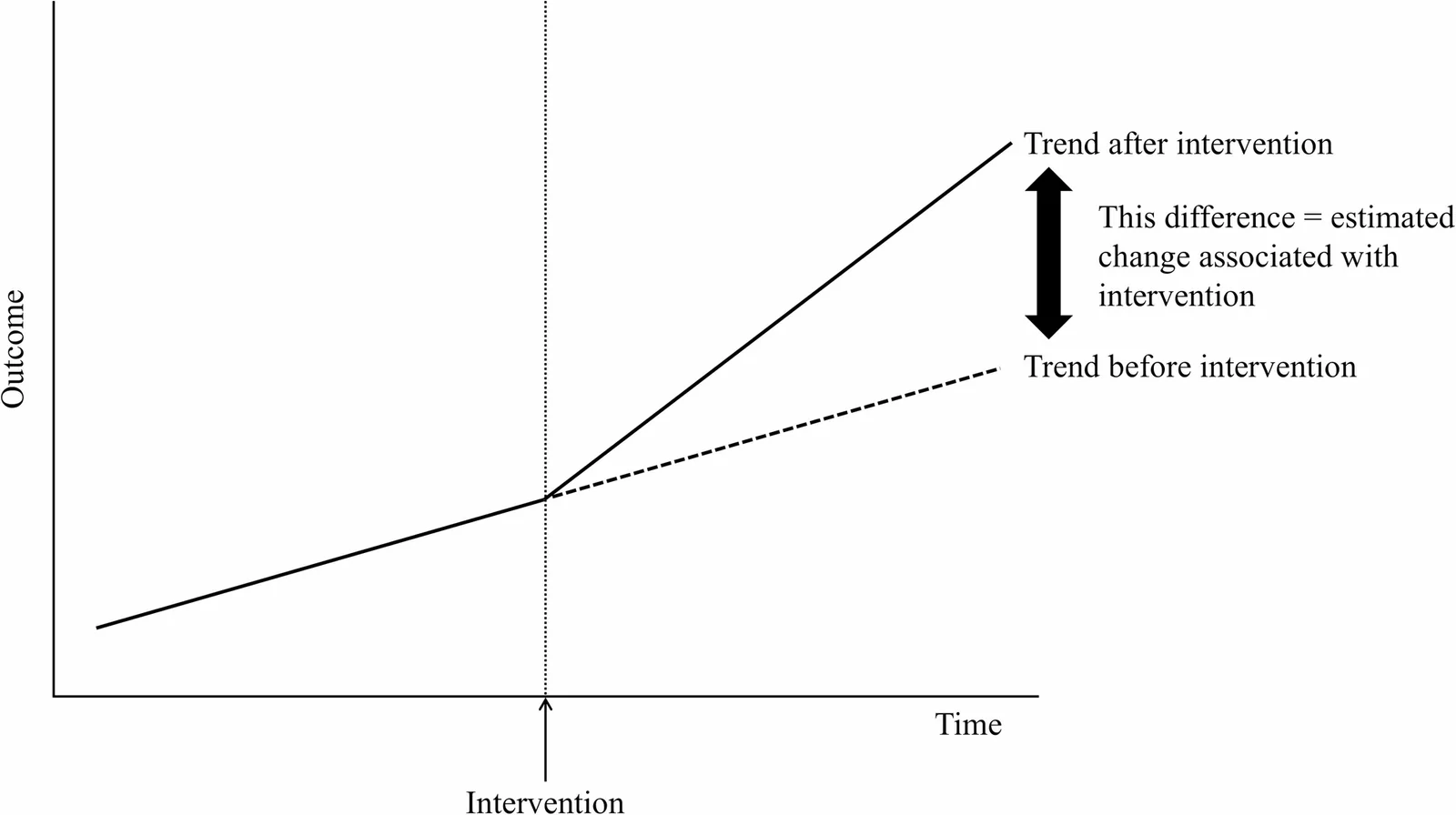
Schematic illustration of the interrupted time-series impact model used in this study. This figure illustrates the assumed slope-change model before and after the intervention point and does not represent observed data

We conducted a chi-squared test to compare the generic dispensing ratio before and after the supply restrictions and used a Mann–Whitney U test to compare medication costs. Furthermore, we assessed changes associated with supply restrictions using ITS in a quasi-experimental design. For the statistical model, we adopted an impact model with slope change, assuming that there was little change immediately before and after the start of supply restrictions but that changes occurred gradually over time [3]. We performed modeling using a generalized linear model with a Poisson distribution (Fig. 1). Statistical analysis was conducted in JMP Pro 16.2 (SAS Institute Inc., Cary, NC, USA). *P*-values less than 0.05 were considered statistically significant.

## Results

We evaluated changes in the generic dispensing ratio and medication costs for quetiapine tablets before and after the start of generic drug supply restrictions. As shown in Table 2, there were a total of 425 prescriptions that included quetiapine before the supply restrictions were implemented, with 19 prescriptions (4.47%) dispensed as branded drugs and 406 prescriptions (95.53%) dispensed as generic drugs. However, after the supply restrictions, 445 prescriptions included quetiapine, of which 146 prescriptions (32.81%) were dispensed as branded drugs and 299 prescriptions (67.19%) were dispensed as generic drugs. The generic dispensing ratio was significantly decreased owing to the supply restrictions (*p* < 0.01).

**Table 2:** Daily branded and generic dispensing ratios before and after supply restrictions

	Branded	Generic	Total
Before(2021.5.1–8.31)	19 (4.47%)	406 (95.53%)	425 (100%)
After (2021.9.1–12.31)	146 (32.81%)	299 (67.19%)	445 (100%)

Pearson’s chi-square test, *p* < 0.0001

**Fig. 2:**
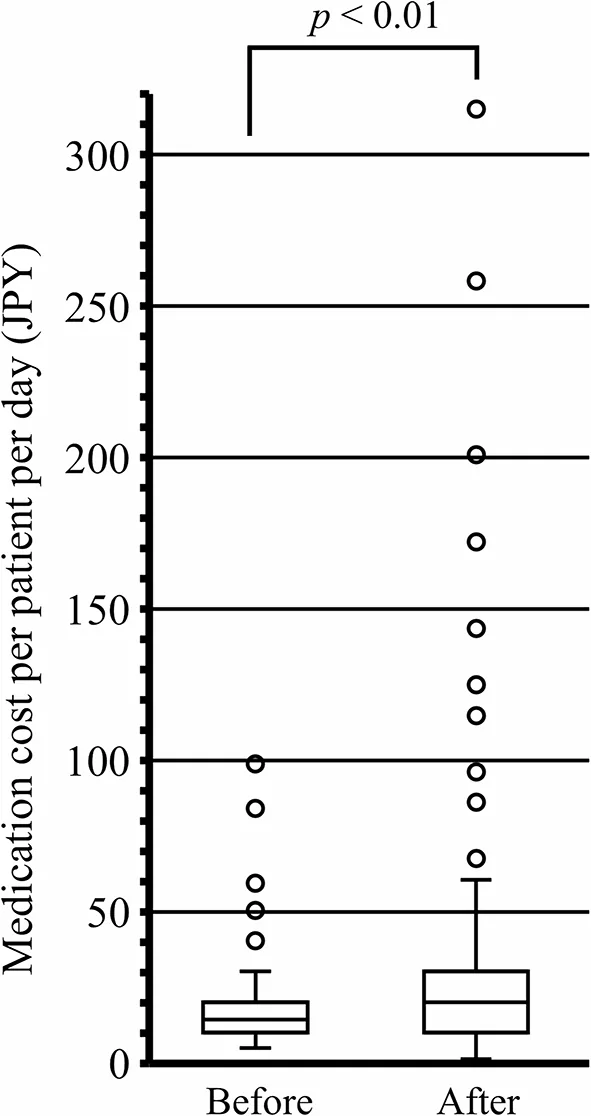
Medication cost per patient per day before and after supply restrictions. (Mann–Whitney U test)

The medication cost per patient per day before and after the supply restrictions is shown in Fig. 2. The medication cost per patient per day was 14.4 Japanese yen (JPY; approximately 0.13 US dollars [USD]/0.11 euros [EUR]) before the supply restrictions (min: 5.1 JPY, max: 98.8 JPY) and increased significantly to 20.2 JPY (approximately 0.18 USD/0.16 EUR) after the supply restrictions (min: 1.3 JPY, max: 315.7 JPY) (*p* < 0.01). Currency conversions were approximate and based on the average exchange rates during the study year.

**Fig. 3:**
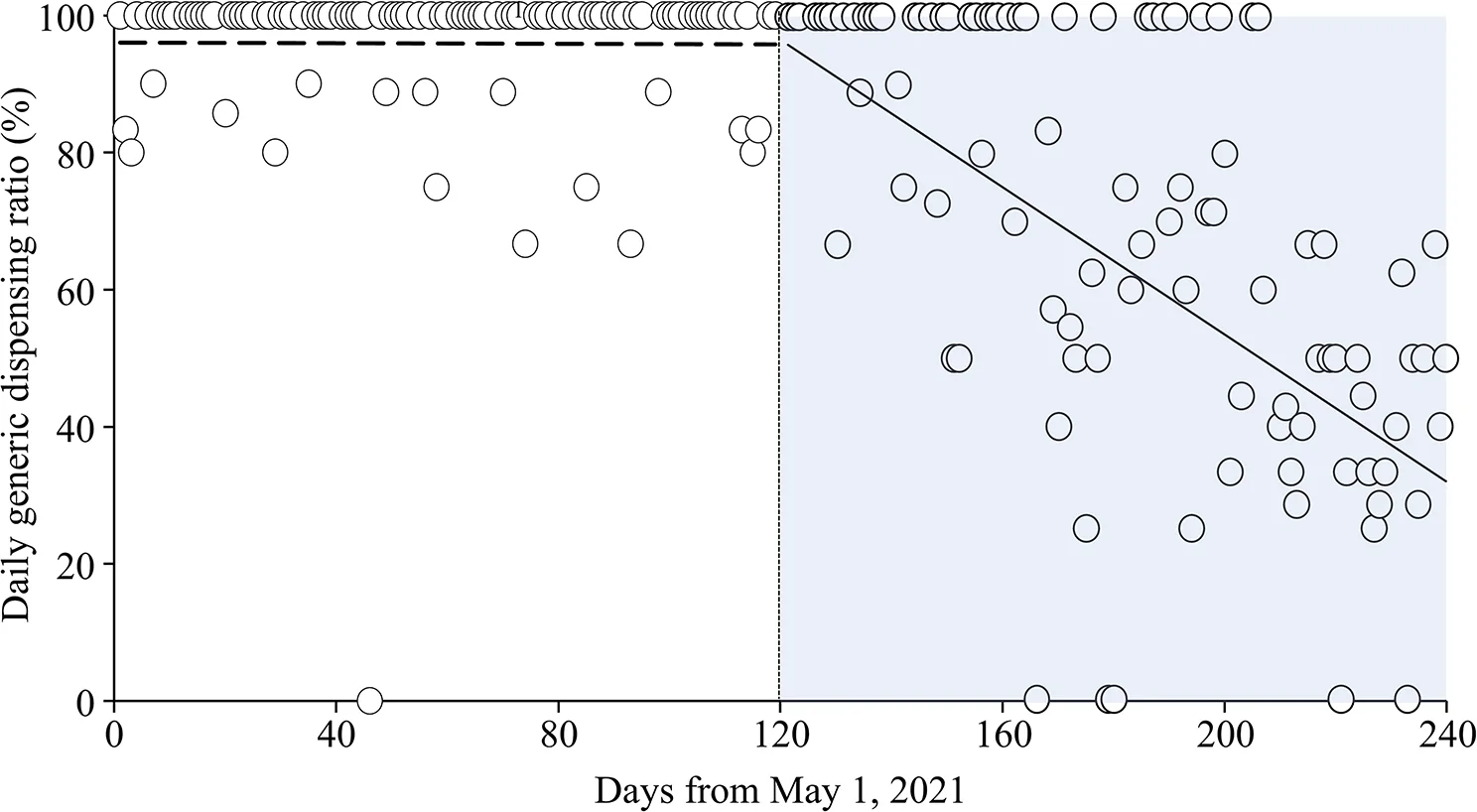
Scatter plot of the daily generic dispensing ratio over time. White background, before the supply restriction period; gray background, after the supply restriction period; dashed line, before trend; continuous line, after trend

**Fig. 4:**
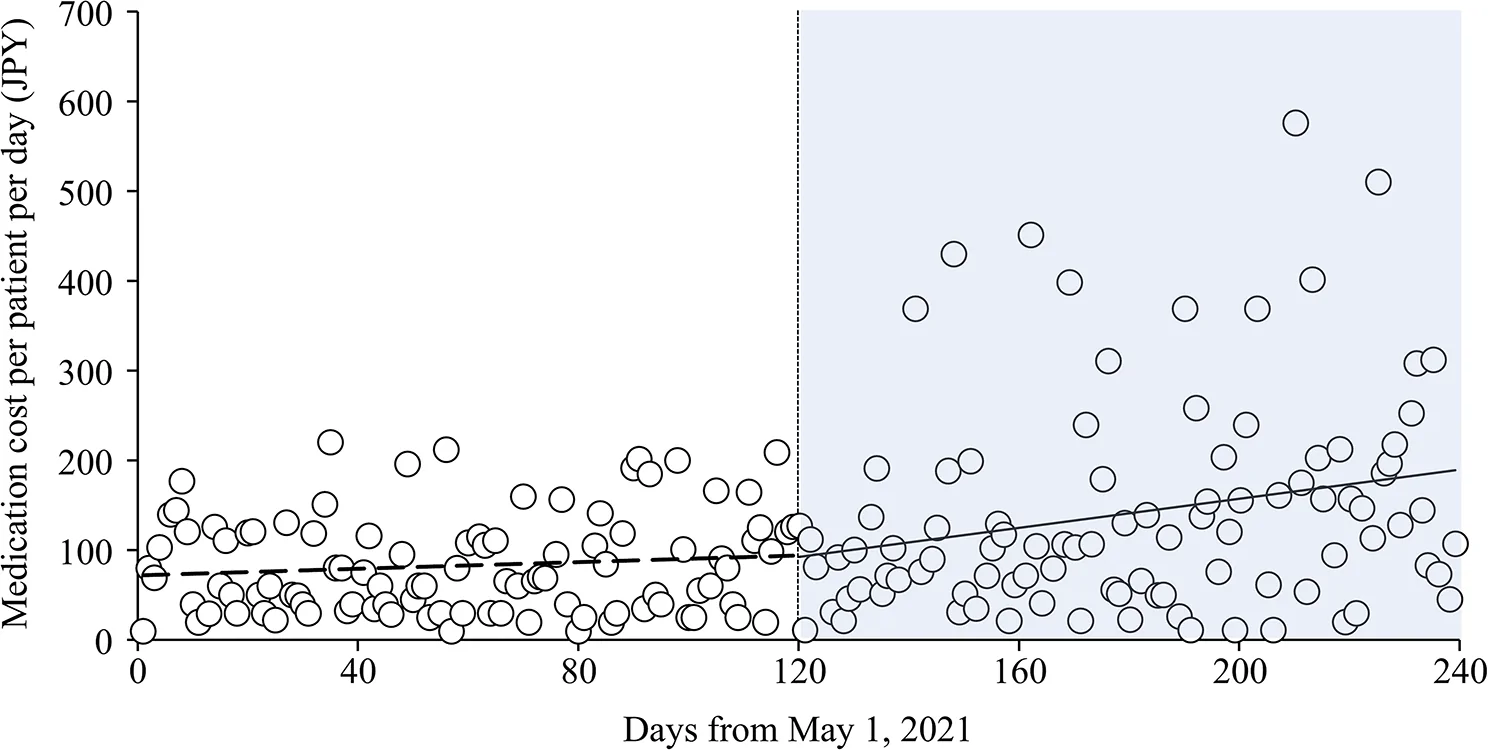
Scatter plot of medication cost over time. White background, before the supply restriction period; gray background, after the supply restriction period; dashed line, before trend; continuous line, after trend

Scatter plots of the generic dispensing ratio and medication cost over time are shown in Figs. 3 and 4, respectively. The results of analysis using ITS with a generalized linear model, assuming a gradual influence owing to supply restrictions, are presented in Table 3. The trend slope of the generic dispensing ratio prior to intervention (i.e., supply restrictions) and the slope change owing to intervention were 0.0006 (95% confidence interval [CI]: −0.0017 to 0.0028, *p* = 0.6297) and − 0.0077 (95% CI: −0.0120 to − 0.0034, *p* = 0.0004), respectively, showing a significant change. The trend slope of medication costs prior to intervention and the slope change owing to intervention were 0.0012 (95% CI: 0.0008 to 0.0017, *p* < 0.0001) and 0.0043 (95% CI: 0.0035 to 0.0051, *p* < 0.0001), demonstrating a significant change owing to the supply restrictions.

**Table 3:** Interrupted time-series regression analysis of generic dispensing ratio and medication cost with supply restrictions

	Generic dispensing ratio		Medication cost	
	Trend (95% CI)	*p* value	Trend (95% CI)	*p* value
Pre-interruption slope	0.0006 (− 0.0017–0.0028)	0.6297	0.0012 (0.0008–0.0017)	< 0.0001
Slope change with interruption	−0.0077 (− 0.0120 – −0.0034)	0.0004	0.0043 (0.0035–0.0051)	< 0.0001

CI, confidence interval

## Discussion

In the present study, we observed changes in the generic dispensing ratio and medication costs for quetiapine tablets before and after generic drug supply restrictions. Furthermore, the interrupted time-series analysis suggested that the effects of supply restrictions became more pronounced over time after their implementation. The supply restrictions evaluated in this study occurred in the broader context of generic drug supply instability in Japan, which was triggered by quality-related problems, including tampering with quality tests by pharmaceutical companies, as described in the Background section. Although the present study did not directly evaluate manufacturing processes, GMP compliance, or quality control issues, our findings suggest that supply disruptions arising from such problems may extend beyond manufacturers and wholesalers to community pharmacy practice.

Previous reports have described the widespread instability of generic drug supply in Japan. According to the investigation by the Federation of Pharmaceutical Manufacturers’ Associations of Japan, as of June 2023, 3882 pharmaceutical items were subject to supply restrictions and supply suspensions, accounting for as much as 22.3% of the total [4]. Izutsu et al. reported that medication manufacturing occurs in two stages: active pharmaceutical ingredient production and formulation, each of which carries a risk of supply shortage [5]. In addition, Izutsu et al. reported that GMP noncompliance and associated quality issues contributed to generic drug shortages in Japan [6]. Kosaka et al. discussed Japan’s generic drug crisis in relation to quality assurance failures, including the case of Kobayashi Kako Co. Ltd., and highlighted its implications for the sustainability of generic drug supply and the Japanese health care system [7]. These previous reports mainly described the scale and background of drug supply instability from regulatory, manufacturing, supply-chain, and health-system perspectives. In contrast, the present study adds pharmacy-level evidence by evaluating actual dispensing data and medication costs for quetiapine tablets in community pharmacies.

ITS analysis is an analytical method for evaluating the effects of interventions by examining changes in temporal trends before and after the intervention. The results of our ITS analysis suggested that the effects of supply restrictions on the generic dispensing ratio of quetiapine tablets and medication costs became more pronounced over time. These findings suggest that prolonged supply restrictions may affect dispensing patterns and increase medication costs for patients.

The Ministry of Health, Labour and Welfare has implemented various measures to ensure the stable supply of medications; however, supply restrictions remain an important issue. These findings suggest the need to consider systems that support the stable supply of medications and reduce the impact of supply restrictions on community pharmacy practice and patient medication costs.

This study has several strengths. First, we used routinely collected dispensing data from multiple community pharmacies, which allowed us to evaluate actual dispensing practices in a real-world setting. Second, we assessed both the generic dispensing ratio and medication costs, enabling evaluation of the influence of supply restrictions from both dispensing-practice and patient-cost perspectives. Third, the interrupted time-series analysis allowed us to evaluate changes in trends before and after the start of supply restrictions rather than relying only on a simple before-and-after comparison. However, this study has some limitations. First, the anonymized dispensing data did not include patient-level demographic or clinical information, such as age, sex, diagnosis, reasons for quetiapine use, occupation, education status, or family background. In routine pharmacy records in Japan, information such as occupation, education status, and family background is not consistently recorded and was not available in the dataset used for this study. Therefore, we could not evaluate patient characteristics or clinical reasons associated with quetiapine use, dispensing patterns, or medication costs. Second, approximately 4000 items were subject to supply restrictions, each with a different timing and duration of restrictions. Therefore, it was challenging to determine the overall influence on health care using the present analytical method. Third, the duration required for a return to the pre-restriction state after the lifting of supply restrictions was not examined; this should be a topic for future investigation. Swift normalization of the supply of quality-assured medications is warranted.

## Conclusion

In this retrospective observational study using routinely collected dispensing data from community pharmacies, generic drug supply restrictions for quetiapine tablets were associated with a decrease in the generic dispensing ratio and an increase in medication costs. The interrupted time-series analysis suggested that these effects became more pronounced over time after the start of supply restrictions. These findings suggest the importance of considering the potential impact of generic drug supply restrictions on dispensing practices and patient medication costs in community pharmacies. Further studies are needed to evaluate whether similar effects occur for other drugs and to assess the broader impact of generic drug supply restrictions on pharmacy practice and health care costs.

## Data Availability

The datasets used and/or analyzed during the current study are available from the corresponding author on reasonable request.

## Abbreviations

CI: Confidence interval
COVID-19: Coronavirus disease 2019
ITS: Interrupted time-series
JPY: Japanese yen

## References

[1] Ministry of Health, Labour and, Welfare J. Roadmap for Further Promotion of Generic Drugs. 2013. https://www.mhlw.go.jp/stf/shingi/2r9852000002yu25-att/2r9852000002zb0m_1.pdf. Accessed 26 Oct 2023.

[2] Ministry of Health. Labour and Welfare, Japan. White Paper on Health and Labor 2023, https://www.mhlw.go.jp/wp/hakusyo/kousei/22/dl/zentai.pdf. Accessed 26 Oct 2023.

[3] Lopez Bernal J, Cummins S, Gasparrini A. Interrupted time series regression for the evaluation of public health interventions: a tutorial. Int J Epidemiol. 2017;46:348–55. 10.1093/ije/dyw098 PMC5407170 27283160

[4] The Federation of Pharmaceutical manufacturers’ associations of Japan. Investigation on Pharmaceutical Supply Situation. (2023.6). http://www.fpmaj.gr.jp/medical-info/results-of-survey/_documents/2023/07/overview.pdf. Accessed 24 May 2026.

[5] Izutsu K, Abe Y, Kurita M, Yoshida H. Shortages of prescription drugs due to compliance and quality issues in Japan. Yakugaku Zasshi. 2023;143:139–52. 10.1248/yakushi.22-00185 36418079

[6] Izutsu KI, Ando D, Morita T, Abe Y, Yoshida H. Generic drug shortage in Japan: GMP noncompliance and associated quality issues. J Pharm Sci. 2023;112:1763–71. 10.1016/j.xphs.2023.03.006 36965844

[7] Kosaka M, Ozaki A, Kaneda Y, Saito H, Yamashita E, Murayama A, Mamada H, Tanimoto T, Jakovljevic M. Generic drug crisis in Japan and changes leading to the collapse of universal health insurance established in 1961: the case of Kobayashi Kako Co. Ltd. Cost Eff Resour Alloc. 2023;21:35. 10.1186/s12962-023-00441-z PMC10230667 37259132

